# Food Insecurity in Individuals with Eating Disorders: A UK-Wide Survey of Impact, Help-Seeking, and Suggestions for Guidance

**DOI:** 10.3390/nu18050852

**Published:** 2026-03-06

**Authors:** Callum Bryson, Jessica Wilkins, Başak İnce, Amelia Hemmings, Carina Kuehne, Daire Douglas, Matthew Phillips, Helen Sharpe, Ulrike Schmidt

**Affiliations:** 1 Centre for Research in Eating and Weight Disorders, Department of Psychological Medicine, Institute of Psychiatry, Psychology & Neuroscience, King’s College London, London SE5 8AF, UK; callum.bryson@kcl.ac.uk (C.B.); jessica.wilkins@kcl.ac.uk (J.W.); basak.ince@kcl.ac.uk (B.İ.); amelia.1.hemmings@kcl.ac.uk (A.H.); carina.1.kuehne@kcl.ac.uk (C.K.); daire.douglas@kcl.ac.uk (D.D.); 2 Department of Psychology, School of Life Sciences and the Environment, Royal Holloway, University of London, London TW20 0EX, UK; matthew.phillips.2023@live.rhul.ac.uk; 3 School of Health in Social Science, University of Edinburgh, Edinburgh EH1 1LE, UK; helen.sharpe@ed.ac.uk; 4 South London and Maudsley NHS Foundation Trust, London SE5 8AZ, UK

**Keywords:** food insecurity, eating disorders, treatment, recovery, help-seeking, clinical guidance

## Abstract

**Background/Objectives**: Food insecurity (FI) is significant and increasing in the UK’s general population. Previous research has linked FI to disordered eating and obesity, yet most of this research is conducted in non-clinical populations in the USA. As such, little is known about the perspectives of people with current or past eating disorders (PwEDs) on the effects of FI on their eating disorder symptoms and treatment in the UK. The current study explores these effects, as well as PwEDs’ experiences of help-seeking for food insecurity and their suggestions for clinical guidance. **Methods**: Data were collected via an online survey (*n* = 337) which included both open-ended and fixed response questions. A mixed methods approach was used for analysis, with a thematic analysis being used for qualitative data. **Results**: Recent FI was related to higher frequency of food restriction and less comfort discussing FI in clinical contexts. Additionally, participants reported that FI exacerbated symptoms and worsened recovery efforts. Help-seeking was generally low among participants. Shame was a barrier for help-seeking, and suggestions for guidance included adaptations to screening and treatment, improving clinician knowledge, and providing practical solutions to alleviate FI. **Conclusions**: FI is a serious public health issue in the UK that has large ramifications for eating disorder maintenance and recovery. Guidance is needed to address FI in clinical practice and reduce shame around FI. Ultimately, however, FI is a systemic issue that will require policy change to be eliminated.

## 1. Introduction

Food insecurity (FI) is defined as the inadequate or unreliable access to a sufficient amount of nutritious food and is often related to poverty or other forms of resource scarcity (e.g., access to water) [1]. FI experiences exist on a continuum from complete food security to moderate (e.g., skipping a meal) and severe forms (e.g., going an entire day without eating) [2]. The recent COVID-19 pandemic has led to an increase in FI globally [3], across countries in both the Global North and South [4,5,6,7]. While rates of FI differ over national contexts, there appears to be a shared vulnerability in certain populations, such as households with low income and children, and individuals with periods of unemployment and mental health diagnoses or disabilities [7,8,9,10]. Post-pandemic rates of FI in the UK are estimated to be around 13.9%, or 7.3 million adults, with severe FI remaining 1.5 times higher now than at the start of the pandemic [11,12]. Importantly, the UK has struggled with exceptionally high FI for some time. The UN’s Food and Agriculture Organization estimated that between 2015 and 2017, on average, 2.2 million people in the UK had at least one experience of severe FI [13], which accounted for around 20% of these cases in Europe [1]. The available evidence suggests that these elevated rates of FI are connected to a wider array of social inequalities and policy decisions [14]. For example, FI has been consistently linked to austerity measures employed by the UK government in response to the 2008 Financial Crisis [15]. Additionally, since late 2021, the cost of necessities in the UK (such as heating, food, and transport) has risen, leading to a so-called cost-of-living crisis where approximately 12% of adults have been struggling financially [16].

The health consequences of FI are wide reaching. Inadequate access to nutritious foods impacts a myriad of physical health conditions, such as anaemia [17], diabetes [18], osteoporosis [19], respiratory diseases [20], chronic pain [21], obesity [22], and malnutrition [23]. For mental health conditions, however, the link with FI is less well understood. Individuals with experiences of FI have significantly higher rates of depression (diagnoses and symptoms) [24,25], as well as an elevated risk of suicidal ideation compared with those without such experiences [26]. Stress and anxiety levels have also been shown to be higher in FI individuals relative to those who are food secure [22,24,25]. Even though it is difficult to make causal inferences about these relationships—as evidence mostly comes from cross-sectional studies—this relationship is likely bidirectional, with FI increasing susceptibility to, and being influenced by, experiences of poor mental health [27].

Attempts to understand how FI impacts eating disorders (EDs) are nascent. EDs are a set of severe psychiatric conditions characterized by pathological eating behaviours [28], such as restriction of food intake, little to no interest in food or eating, binge eating, and/or engaging in compensatory behaviours (e.g., over-exercise or self-induced vomiting) to control weight and shape [29,30]. Notably, EDs have some of the greatest mortality and morbidity rates of any mental health condition. This underscores the importance of understanding risk factors that affect their development, maintenance, and relapse [31]. The available evidence indicates that, relative to food secure adults, FI is associated with a higher prevalence of: eating, weight and shape concerns [32], ED behaviours (such as objective binge eating and compensatory fasting) [33,34], and EDs like binge eating disorder (BED) and bulimia nervosa (BN) [35,36,37].

There are a number of mechanisms that may drive disordered eating in individuals with FI. First, FI is known to create a stressful preoccupation with food (e.g., via anxiety around when food can next be accessed, around choosing between food and other necessities, or due to shame around how food is acquired) which is thought to lead to other ED behaviours [38]. Second, stress may exert an indirect impact on the relationship between FI and EDs via epigenetic influences on the expression of key genes in vulnerable populations [39]. Third, binge behaviours in FI populations may arise from the ‘feast/famine’ cycle of food availability [37]; in times of relative dearth, food intake may be restricted by necessity or with intention (a known risk factor of binge eating) [40]. Binges may then follow on from periods of restriction as an emotion regulation tool in response to stress experienced by FI [41] or due to the compounding effects of weight stigma and body shame for those in larger bodies [42]. There has been less focus on understanding the link between restrictive ED behaviours and FI in the literature, and as a consequence, there is a less cogent explanation for how FI may lead to illnesses like anorexia nervosa (AN), other than the generic mechanisms of stress [37], guilt, and eating [43]. Moreover, most of the focus in this literature has been on how FI contributes to the aetiology of EDs, with there being a relative absence of research surrounding how FI experiences may contribute to their persistence and relapse risk.

Although evidence indicates that FI and EDs are related, most studies have been conducted in non-clinical samples in the USA, so their comparability to ED populations and other national contexts is unclear [44]. Emerging evidence suggests a similar relationship in a UK sample [45], and a comparison with other national contexts has found the association between FI and binge/purge symptoms to be similar across the UK, USA, Australia and Canada [46]. According to a recent survey, ED healthcare professionals in the UK perceive FI as a growing issue for their patients, but feel they lack practical guidance and knowledge to address FI challenges in their clinical practice [47]. Lived-experience perspectives are valuable for understanding the role of FI in the pathogenesis of EDs and its impact on treatment; however, they have been largely underutilized [37]. Qualitative research from the US has shown that FI is implicated in the maintenance of ED symptoms and impairs patients’ ability to engage in treatments, but is largely unaddressed by clinicians [48]. Similarly, evidence from Canada suggests that FI (and other social determinants of health) impedes access to and engagement with first-line ED treatments [49]. However, it is unknown how applicable these findings are to the ED treatment and service context of the UK and the National Health Service (NHS) in light of the recent cost-of-living crisis.

The current study used a mixed methods approach via an online survey in the UK to (a) quantify the experiences of FI, cost-of-living issues, and help-seeking in people experiencing a diagnosed or suspected ED, and (b) qualitatively explore their perspectives of the role that FI plays in the aetiology, maintenance, and relapse risk of their illness, as well as their suggestions for future clinical guidance. To achieve this, the following research questions were devised:

1. To what extent do people with a past or present ED (PwEDs) experience FI and other cost-of-living issues in the UK? How do PwEDs view FI and the cost of living in relation to the development, maintenance, and treatment of their ED?
2. To what extent do PwEDs seek help from healthcare professionals regarding their FI experiences? What factors facilitate or impede this help-seeking?
3. What suggestions for guidance (if any) do PwEDs have for how FI can be managed in healthcare settings?

## 2. Materials and Methods

### 2.1. Participants and Procedure

Ethical approval for the study was attained via the King’s College London Research Ethics Committee (Review Reference: HR-22/23-34804). Eligibility criteria included being at least 16 years of age, residing in the UK, and having current or past ED or disordered eating behaviours. Responses to the online, cross-sectional survey were collected between June 2023 to July 2024, and the survey was advertised online via the research team’s website (https://edifyresearch.co.uk), a reputable ED charity in the UK (BEAT Eating Disorders, Norwich, UK; https://www.beateatingdisorders.org.uk/), and social media (e.g., X (formerly Twitter), Bastrop, TX, USA and Instagram, Menlo Park, CA, USA). These advertisements made clear the study’s eligibility criteria, as well as the aim of the study to “explore the effects of rising living costs, food insecurity and poverty on eating disorder symptoms and treatment”.

**Figure 1:**
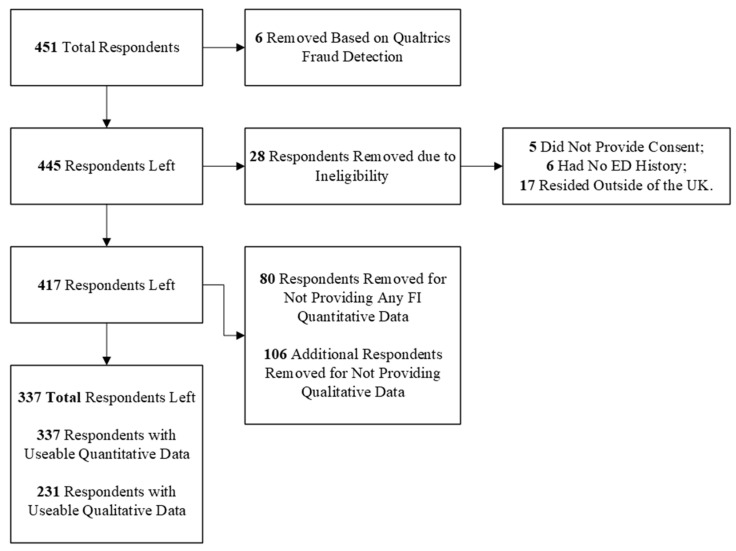
Flowchart of participants.

There were 451 total responses to the survey. Prior to analysis, the data were cleaned to remove responses rated as a high probability of being fraudulent by Qualtrics, as well as responses from ineligible participants or those who stopped the survey prior to answering any FI questions (Figure 1). After data cleaning, there were 337 respondents (92.6% female) between the ages of 16 and 67 who provided some analysable data. Within this sample, 263 had previous diagnoses of at least one ED, while 70 indicated that they experience(d) disordered eating behaviours, and 4 preferred to not report if they had received a diagnosis. As participants may have been diagnosed with multiple EDs over the course of their life, they were able to select all applicable diagnoses they had received.

Participants self-selected to complete the survey after seeing the study advertisement online and clicking on a link to a Qualtrics survey (https://www.qualtrics.co.uk, Provo, UT, USA). At this point, they were also provided with the information sheet. After reviewing this, participants provided their consent and then filled out a brief screening question to check eligibility where, if eligible, they then went on to answer the survey questions. Data were retained from participants who provided partial responses to the survey. Lastly, previous research demonstrates that there are significant barriers to seeking help for an ED, with access to treatment for PwEDs being concerningly low [50,51]. As such, responses from all participants were included in the analyses, regardless of diagnostic status, to decrease the chances of replicating these biases.

### 2.2. Materials and Measures

#### 2.2.1. Survey

Survey questions covered demographics, ED history, food insecurity experiences, other cost-of-living issues, food insecurity and EDs, ED symptoms, and additional thoughts or comments. Supplementary Materials File S1 contains the entire survey. For the sake of brevity, the full questions and responses are not replicated in this section. Definitions of FI at the beginning of the survey came from a 2019 report by the UK government’s Environmental Audit Committee [1]. Most of the survey questions were developed specifically by the authors based on their previous survey with ED clinicians [47]; questions adapted from established sources are listed below.

#### 2.2.2. Food Insecurity Questions

Experiences of mild to severe food insecurity were measured using questions adapted from The Food Foundation’s (a UK-based charity) Food Insecurity Tracking surveys [52] which are based on the US Department of Agriculture’s Adult Food Security Survey [53]. Participants were asked to dichotomously select which of the following aspects of food insecurity they had experienced in the past month: “Been worried about accessing or affording enough nutritious food”, “Had less nutritious or balanced meals because you couldn’t afford or get access to food”, “Had smaller meals than usual or skipped meals because you couldn’t afford or get access to food”, “Ever been hungry but not eaten because you couldn’t afford or get access to food”, “Not eaten for a whole day because you couldn’t afford or get access to food”, or “None of the above”. Other FI questions exploring the relationship between FI and ED and recovery were created for the purposes of the survey. Respondents only answered these questions if they indicated an experience with FI.

#### 2.2.3. Cost-of-Living Experiences

Participants were also asked about their wider cost-of-living experiences [47]. Participants rated the day-to-day impact of several cost-of-living experiences on a scale of 0–100 (0 being no impact). Cost-of-living experiences included (a) rising energy costs, (b) affordability of clothes, (c) rising transport costs, (d) reliance on zero/low hour contracts, (e) the ability to do other, non-food-related activities important for recovery/well-being, and (f) taking time off work to focus on recovery. Participants were also asked an open-ended question to assess the general impact of the cost-of-living crisis on their daily lives.

#### 2.2.4. Eating Disorder Diagnostic Scale

The Eating Disorder Diagnostic Scale (EDDS) for the DSM-5 was included at the end of the survey [54,55]. Composite scores of ED symptoms were used to generate probable diagnoses for participants (i.e., AN, BN, BED, or other specified feeding or eating disorder (OSFED)) [55]. Atypical AN was subsumed under AN, while OSFED included purging disorder and night eating syndrome. A global score of ED symptoms was also derived using a previously established methodology to combine the raw scores of all relevant items [55,56]. Of the 337 total respondents, only 203 provided responses to all of the EDDS items, and thus the probable diagnosis was only computed for these participants. For 134 participants, the probable diagnosis was not computed due to missing responses to some/all EDDS items.

### 2.3. Statistical Analysis

#### 2.3.1. Quantitative Analysis

Data were imported and analysed in SPSS Version 29.0.2.0 (IBM, Armonk, NY, USA). Descriptive statistics were computed for key variables and then participants were split into two groups: those with and without recent experiences of FI. Participants were placed in the former group if they self-reported at least one experience of FI in the past month, while the latter group was comprised of participants who self-reported no experience of the FI items. A liberal criterion was used here to encapsulate the most general experience of FI as possible due to the exploratory nature of the research. Analyses at different thresholds (e.g., reporting 3 or more FI items to be in the recent FI group) were not conducted. Inferential statistics (*t*-tests) were used to investigate whether recent experiences of FI were related to differences in confidence discussing FI in a clinical setting, the impact of cost-of-living experiences, ED symptoms, and probable ED diagnosis. Non-parametric tests, like the Mann–Whitney U test, were used where assumptions for parametric tests were violated. A point-biserial correlation was also conducted to assess the strength of the relationship between FI experiences and ED symptoms. Due to the exploratory nature of the research, a priori power analyses were not conducted. Instead, post hoc power analyses and Cohen’s d effect size were calculated in G*Power Version 3.1.9.7 (https://www.psychologie.hhu.de/arbeitsgruppen/allgemeine-psychologie-und-arbeitspsychologie/gpower; accessed on 22 August 2025, Heinrich Heine University Düsseldorf, Düsseldorf, Germany) using its manual. For Mann–Whitney U tests, the effect size *r* was calculated using the following formula:

$$r=\frac{Z}{\sqrt{N}}$$

where *Z* is the standardized difference between the two groups and *N* is total sample size. Partial responses were retained from participants and included in the analyses to maximise the power for each variable. Probable diagnosis was analysed dichotomously (No vs. Any diagnosis) using a chi-square test and categorically at five levels (No Current ED Diagnosis, Anorexia Nervosa, Bulimia Nervosa, Binge Eating Disorder, OSFED) using a Fisher–Freeman–Holton Exact test. Odds Ratios and Cramer’s V were computed for the tests, respectively.

#### 2.3.2. Qualitative Analysis

The qualitative data obtained from the online survey were analysed using Braun and Clarke’s phases for inductive thematic analysis [57]. Two authors (Bİ, JW) independently coded 100% of the responses and the first author (CB) coded 50% from each question. Researchers (Bİ, JW, CB) then met to search the data for themes, refining them in an iterative process until all themes and subthemes expressed unique aspects of the data. Phase 3 saw the initial generation of 8 themes, which were later reduced to 6 in phases 4 and 5. The researchers then selected appropriate quotes to illustrate each theme/subtheme and created a thematic map to illustrate themes and subthemes.

## 3. Results

### 3.1. Quantitative Results

In the current sample, just over two-fifths (41.8%) of the participants reported experiencing at least one indicator of FI in the past month, with 46 participants (13.7%) endorsing three or more FI items, a marker of low food security. Only 11% of participants reported being asked about FI in healthcare appointments and just 7.7% ever sought help for their FI. Table 1 and Table 2 summarise the sample demographics and descriptives, respectively.

For ED symptoms and probable ED diagnosis, those with recent FI experiences reported engaging in significantly higher rates of fasting to maintain weight and shape, and excessive exercise. No differences emerged between the groups for any other ED symptoms, nor probable diagnosis (see Table 3 and Table 4). Effect sizes were small, and only the finding for fasting had sufficient power. Further correlational analysis revealed no significant relationship between FI experiences and global ED symptoms (*r*_pb_ = −0.099, *p* = 0.158).

**Figure 2:**
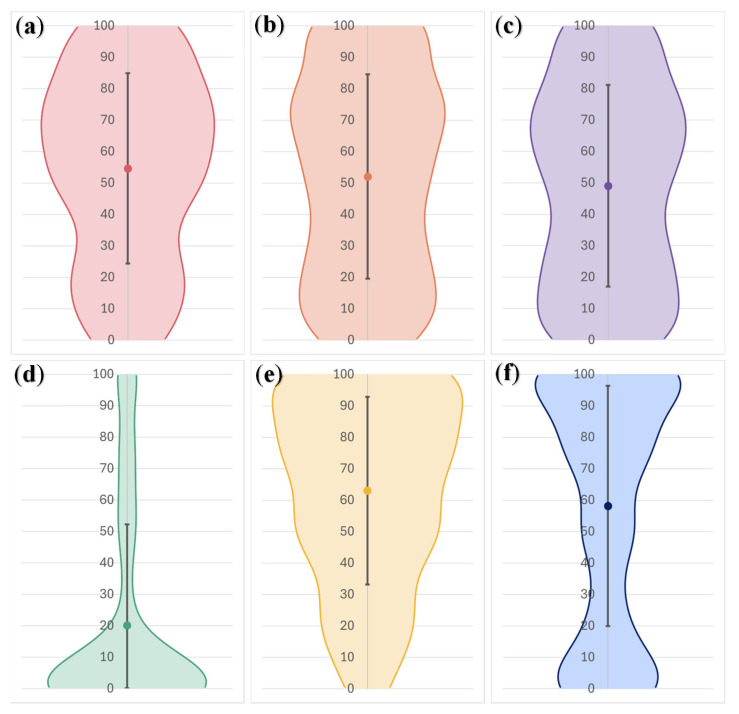
Participant ratings of the impact of several cost-of-living experiences on their day-to-day lives (0 = no impact, 100 = a great deal of impact). (**a**) Rising energy costs; (**b**) affordability of clothes; (**c**) rising transport costs; (**d**) reliance on zero-hour/low-hour contracts; (**e**) ability to do other, non-food-related things important for recovery/well-being; (**f**) taking time off work to focus on health and well-being/recovery.

On average, participants espoused neither comfort nor discomfort with the notion of discussing FI with a healthcare professional during an appointment (*M* = 52.53, *SD* = 32.38, Range = 0–100). However, those with recent experiences of FI were significantly more likely to report discomfort discussing FI in this setting than those without recent FI experiences (see Table 4). Cost-of-living experiences, on average, were perceived to have a moderate impact on participants’ day-to-day lives for all experiences, bar the reliance of zero-hour or low-hour contracts, which was relatively unimpactful on participants (see Figure 2 for illustration). Significantly higher day-to-day impact was reported by participants with recent FI experiences on cost-of-living domains related to increases in the cost and affordability of energy, clothing, and transport, as well as the ability of participants to partake in non-food-related activities important for recovery, relative to participants without such experiences (Table 5). Small to moderate effect sizes were observed. All non-significant effects lacked adequate power.

### 3.2. Qualitative Results

**Figure 3:**
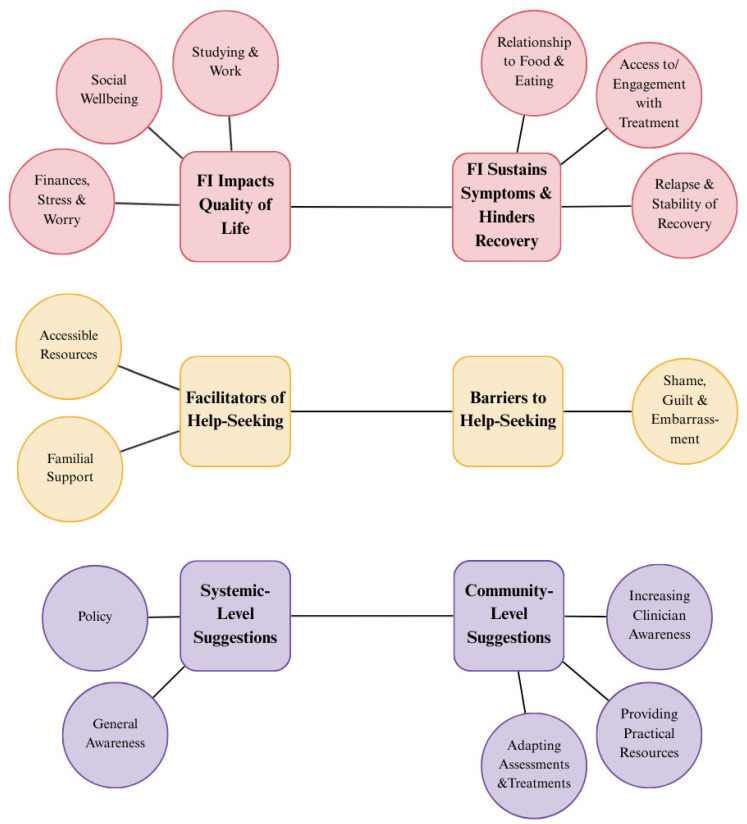
Thematic map. Themes are represented by squares, and the subthemes under them are represented by connected circles. Colours relate to the research question listed at the end of the introduction which are addressed by each theme. Circles in red relate to question 1 (interaction between FI and EDs), circles in yellow relate to question 2 (experiences of help-seeking), and circles in purple relate to question 3 (suggestions for guidance).

Six themes emerged from the qualitative analysis: (1) FI sustains symptoms and hinders recovery; (2) FI impacts quality of life; (3) facilitators of help-seeking; (4) barriers to help-seeking; (5) community-level suggestions for guidance; and (6) systemic-level suggestions (Figure 3). Shame was a recurring topic that was common in responses to most questions and was related to each theme to a certain degree. Here, the following acronyms will be used for brevity: PD = previously diagnosed, ND = not previously diagnosed, UD = unknown diagnosis. Demographics are provided alongside illustrative quotes to describe the participant each quote comes from. Lastly, 231 participants provided useable responses to at least one of the qualitative questions included in the analysis. However, the number of responses to each question was variable, with some participants skipping over or providing responses that did not answer the question (e.g., if asked “How did X…”, they answered “No”.). Appendix A, Table A1 shows the total number of useable responses for each question.

#### 3.2.1. Theme: FI Sustains Symptoms and Hinders Recovery

There were three subthemes under this theme: Relationship to Food and Eating, Access to/Engagement with Treatment, and, Relapse and Stability of Recovery.

Some participants described how childhood experiences of FI contributed to their ED development, while others reported that their ED preceded any experiences of FI. For example, P421 (29, F, White, PD) expressed that they felt “*like this [FI] contributed to, at times, an unhealthy attitude to food and having to finish eating even when I was full as a child. It warped the way I viewed food and eating and probably had an effect when I started to develop an eating disorder.*” Difficulties spending money on food was also described, particularly as participants felt undeserving of food: “*When I do spend money on food, it makes me feel really anxious. I’m constantly worrying about whether I ‘deserve’ to buy food.*” (P30, 18, F, Asian, PD). Eating behaviours, such as restricting food intake, binge eating, and hoarding food, were also described by participants as being impacted by FI. P156 (22, NB, White, PD), for instance, revealed that “*It [FI] has become an excuse for me not to eat, and to buy less food… it gives me a double sense of pride when I don’t eat; I successfully fasted which means I neither gained weight, nor spent money*”. P421 also described how the financial strain of binge eating episodes contributed to feelings of stress and in turn future binges: “*Bulimia is an expensive illness, when I’ve just been paid and can afford to I often spend a lot more on food, especially during binge episodes and it leaves me without the ability to buy nutritious foods later. The anxiety then leaves me more likely to binge again in future… so it’s much harder to break the cycle.*”

Participants also noted that FI and the cost of living had a negative impact on their ability to access treatments. Generally, undertaking recovery was noted to be a “*significant time commitment*” which “*financially… can be challenging.*” (P150, 34, M, White, PD). Physical challenges of accessing treatment were also described by P258 (16, F, White, PD) who stated “*Petrol costs make it incredibly difficult for travelling to my inpatient unit (170 miles away).*” Once in treatment, participants perceived that FI could still be a barrier to engaging with treatment protocols. Specifically, P169 (22, F, Mixed, PD) reported ambivalence towards engaging in meal plans due to their financial cost: “*I know that I need to spend more money on food so that I am able to follow my meal plan, but I can’t really afford to nor do I want to because it feels like a waste of money to spend what little I have on food*”. The inability to comply with these treatment demands was also attributed to a stagnation in recovery: “*It’s [FI] stalling my treatment because I’m always so tired, cold and nauseous (through either hunger or eating food not really fit for consumption) and I can’t justify buying things that, for example, my dietitian wants me to introduce.*” (P174, 45, F, White, PD).

Lastly, for some participants, FI was perceived as increasing their risk of relapse. For example, P59 (40, F, White, PD) explained how “*Although I see myself as having recovered from my eating disorder (*i.e., *I rarely purge these days), the cost of food brings food back to the front of my mind or it’s constantly there despite working hard for years to reduce my obsession with it.*”. For others, the increase in food prices triggered the re-emergence of restrictive eating patterns, as P55 (26, F, Mixed, ND) noted, “*I feel the stress around affording enough nutritious food has highlighted my stress around eating cheap ‘unhealthy’ food and this has given me an excuse to start restricting my intake again*”. This increased risk of relapse was even present when participants had been in recovery for longer periods of time: “*[The] current cost-of-living crisis has caused me to relapse after 3 years in recovery as it’s more economical to eat less, which in turn has triggered my guilt around food.*” (P127, 26, NB, White, PD).

#### 3.2.2. Theme: FI Impacts Quality of Life

Three subthemes emerged from this theme: Studying and Work, Social Well-being, and Finances, Stress, and Worry.

Experiences of FI had a wider impact on other aspects of participants’ lives. Studying and working life were often cited as being negatively impacted by FI: “*Previously I have had to skip meals due to lack of money and would instead have a nap at meal times instead of [eating] food. I was at university at the time and struggling to get by on my student loan and rising cost-of-living. This definitely affected my ability to concentrate and focus on my work.*” (P424, 23, F, White, ND). Disruption to participants’ education/occupation was particularly difficult where it required physical exertion or concentration: “*[I’m]* ‪*less able to do my job well as it is active and has some risk so needs high alertness and energy levels for long periods of time.*” (P28, 30, F, Mixed, PD). Struggles with FI were also described as more acute when participants ability to work was disrupted by their ED, as P176 (43, F, White, PD) revealed, “*[I] need to eat more food in recovery and can’t afford this because I am off sick from work*”.

Participants also perceived their social well-being as worsened by experiences of FI. For some, FI compounded anxiety around social eating. P269 (26, F, White, PD), for instance, stated, “*It [FI] has also limited my social interaction with people as I avoid social events where food is involved due to the costs, as well as the anxiety surrounding socialising and eating itself*”. Some participants also described a trade-off between affording food and socialising, such as P127 (26, NB, White, PD), who “*Started buying less food in order to still be able to enjoy life and have fun experiences (*i.e.*, travelling to see friends, going to social events, keeping up hobbies)*”. Experiences of isolation were also perceived to be detrimental to participants’ recovery efforts, as illustrated by P250’s (29, F, White, PD) response, “*I use it [the cost-of-living crisis] as an excuse not to do certain things like go out for meals as it increases my anxiety- I worry about money so don’t do things that will help challenge my eating disorder. As a result I do less and isolate myself more*”. Likewise, struggles with recovery compounded difficulties with socialising: “*It [FI] has slowed down my progress, I can feel really poorly some days and by not improving or trying new food, it means my relationship and friendships are impacted as it is not easy to socialise if any form of meal is involved*” (P238, 27, F, White, PD).

Lastly, experiences of FI contributed to feelings of stress and worry in participants’ everyday lives. For example, participants experienced stress from increasing food prices, “*I am stressed, anxious, and depressed by the thought of eating nutritious meals as the shock of prices going up every time I go the shops is too much.*” (P111, 40, F, Asian, ND). Participants also described other stressors related to FI, such as having to choose between accessing food over other household necessities “*I had to choose whether to pay my rent or eat and unfortunately often times rent would come before health*” (P374, 24, F, White, PD) or the need to prioritise food access for children in a household “*I will do anything for my kids even if that means starving myself*” (P216, 27, F, White, PD). Additionally, participants described increased stress as particularly detrimental to their ED. For P105 (24, F, White, ND), engaging in restrictive eating behaviours was used to manage the stress from both FI and their ED: “*it just makes me feel like spending more on food gives me stress and gaining weight makes me stressed so it’s easier to control both by eating less*”.

#### 3.2.3. Theme: Facilitators of Help-Seeking

Two subthemes were identified for this theme: Accessible Resources and Familial Support.

Positive experiences of help-seeking usually occurred where participants were provided with accessible resources. For P358 (20, F, White, PD), access to free food at school and validation from their GP was key to their help-seeking experience: “*My GP is amazing and made me feel less embarrassed. I got free food at school which was good. It was discreet and also packaged so I could take it home for my sister/mum and brother*”. Access to free, discrete food at work was also described by P79 (26, NB, White, ND) who said, “*at my induction with a major supermarket. I wasn’t able to afford a meal deal for lunch as I had unplanned expenses, but their staff canteen had free pot noodles/pasta mug shots and other breakfast and lunch-type foods for staff to utilise in times of food insecurity*”.

Participants reported informal help-seeking via family members, for example, P355 (28, F, White, PD) stated, “*I asked my mum to help me financially to afford to get myself a variety of ‘safe’ snacks and meals*”. However, having to ask for help could also cause shame or guilt. P439 (47, F, White, ND), for instance, described guilt about burdening family, “*I’ve had to ask my mum for money at the end of lots of months for food for the last few days before payday. I feel ashamed and embarrassed. My mum helps me willingly but I’m a grown up and shouldn’t be putting her in this position.*” (P439, 47, F, White, ND). For others, like P26 (26, F, White, ND), shared experiences of struggling with FI and the cost-of-living crisis reduced shame and facilitated help-seeking, “*I wasn’t scared of stigma or telling anyone, as I felt all of my friends and colleagues were going through the same and no one could really afford to heat their entire house/flat*”.

#### 3.2.4. Theme: Barriers to Help-Seeking

Shame, Guilt and Embarrassment was the only subtheme to surface from this theme.

Feelings of guilt, shame, and embarrassment were widely noted barriers to help-seeking. P358 (20, F, White, PD), who described positive experiences with accessing food in school, also mentioned other attempts to ask directly for food left them feeling “*weak*” and “*disgusting*”. Another participant, P176 (43, F, White, PD), detailed how embarrassment around FI prevented them from seeking help from their treatment team: “*I wanted to ask my Eating Disorder team for a referral to a food bank but I was too embarrassed to do so. Also my eating disorder told me this was greedy*”. Resource use was also accompanied by marked guilt and shame, as said by P20 (22, F, White, PD): “*I am care experienced, I spoke to my leaving care workers, who were able to give me an emergency voucher for my local supermarket. I felt guilty for asking for help and felt guilty spending the voucher on food that I liked*”. Participants also worried about depriving others of resources, “*[I received an] Emergency food bank referral. Feel guilty for using it when other people need it*” (P28, 30, F, Mixed, PD), and were concerned about not fitting the intended service demographic, “*when I go to the foodbank, I feel a sense of shame because I think to myself “what the fuck am I doing here? I usually have money to eat! the foodbank is only for people who are dirt poor and I’m on maximum disability benefit!!”*”.

#### 3.2.5. Theme: Community-Level Suggestions for Guidance

There were three subthemes that surfaced under this theme: Adapting Assessments and Treatments, Providing Practical Solutions, and Increasing Clinician Awareness.

A majority of participants’ suggestions for clinical guidance were related to creating adaptations to assessment and treatments. In terms of assessments, participants felt that discussions about FI should be standard for all patients, as illustrated by P382’s (20, F, White, PD) response*:* “*There should be a discussion with each patient at start of the treatment to see if this is an issue or not*”. They additionally wanted clinicians to take initiative when starting these discussions: “*Clinicians could be given some prompt questions to approach the subject with kindness and to make the patient feel less anxious or shameful*” (P267, 39, F, White, PD). Preferences for assessment format varied among participants. P267 preferred questionnaires, “*perhaps a discreet questionnaire could be developed to be filled in*”, while other participants found these to be impersonal: “*not* via *a questionnaire that’s then given to the GP as this can feel a bit impersonal and intrusive*” (P29, 16, F, White, PD). While many participants endorsed the idea of clinicians starting a conversation about FI, others disagreed: “*Prefer not to [discuss FI] as it’s embarrassing*” (P93, 40, F, White, PD).

For treatment, participants noted that meal plans were sometimes untenable on a budget, and so they wanted clinicians to be able to provide plans that were more suited to participants’ financial situations. For example, P26 (26, F, White, PD) suggested the clinicians could provide “*weekly varied meal plans with stretched budgets (so I can go food shopping once a week or so and thus have a better overview of my expenses)*”, as well as “*ideas for smaller, simple in-between meals to avoid going a day without eating*”. This sentiment was echoed by P50 (26, F, White, PD) who also recommended that guidance include information on “*low cost, nutritious recipe ideas for recovery e.g., using tinned foods, advice on how to mix up food brands—this is really hard in recovery and makes you rigid but some foods that are comfortable are more expensive now*”.

Outside of accommodations to assessment and treatment, participants wanted healthcare professionals to provide practical, useable advice for resources to receive food. For example, participants disliked the notion of clinicians offering them nothing but commiserations: “*[It’s] helpful if they have something they offer. So not just say that it must be hard,* etc.*, but actually provide practical solutions (*e.g.*, food bank locations).*” (P52, 29, F, White, ND). Generic advice was also something participants wanted clinicians to avoid, as P19 (27, F, White, ND) shared, “*I would probably avoid recommending low cost foods* e.g.*, pasta, rice, pulses* etc. *as this is information parroted back to people whenever issues of poverty/CoL [cost-of-living] crisis are raised and it can feel a bit condescending (like of course I’m trying to shop with a budget and aware of cheaper ‘staple’ items)*”. This generic advice was also sometimes described as being detrimental to ED symptoms: “*sometimes cooking from scratch, batch cooking and buying things in bulk are all not possible or advisable due … exacerbating eating disorder symptoms such as bingeing.*” (P99, 40, F, White, PD). Additionally, participants wanted healthcare professionals to provide this information or for it to be freely available in clinical settings, rather than simply receiving encouragement to look for this information themselves, as P108 (31, F, White, PD) detailed, “*Real resources and the meaningful information to receive them—telling someone to google the resources in their area isn’t actually helpful. Making this information, when possible, visibly accessible—much like signs to remind people to get their flu shots, encourage people to speak to their doctors if they are struggling with food insecurity or the cost-of-living because these are valid health questions/concerns*”.

Lastly, participants wanted healthcare professionals to be more knowledgeable and aware about FI and to work towards destigmatisation. P241 (17, F, White, PD), for instance, stated that “*…if this concept [financial struggles] was more respected and talked about, people may not feel as ashamed when admitting that they are having money problems*”. Participants identified the need to raise awareness of how different diagnostic and social characteristics intersect with experiences of FI and recovery, such as those experiencing binge behaviours or ARFID. P197 (21, NB, Black, PD), for instance, suggested that “*clinicians should be aware of how food insecurity can affect several types of eating disorders, so not just restrictive but also binge eating*” (P197, 21, NB, Black, PD). Single parent households were also identified as being particularly vulnerable: “*Make the effort to understand that my recovery in a single parent, working class family is not the same as the recovery of somebody who lives in a middle-class family.*” (P30, 18, F, Asian, PD). Participants also identified the need for improved awareness for healthcare professionals, especially GPs, in understanding FI from lived experience perspectives: “*A guide on how to approach the subject [FI] and how to get across the seriousness of our concerns. I think GPs especially don’t have much training in eating disorders so don’t understand the underpinnings of the illness.*” (P221, 28, F, White, PD). Empathy and compassion from healthcare professionals was also consistently cited by participants: “*Clinician’s [need] to be emphatic, maybe attend some training on how to approach the topic with someone with an ED*” (P70, 28, F, White, PD).

#### 3.2.6. Theme: Systemic-Level Suggestions for Guidance

Two subthemes emerged under this theme: Policy and General Awareness.

Firstly, participants acknowledged that FI was a systemic issue that would require changes to policy to resolve. For example, it was suggested by P424 (23, F, White, ND) that guidance “*shouldn’t only focus on the public and clinicians… it should… make them [the government] aware of the negative consequences of the rise in cost-of-living on food prices, people’s lives and their health. Focusing just on clinicians and [the] public isn’t going to tackle the heart of the problem*”. P156 (22, NB, White, ND) linked “*the CoL crisis, food insecurity, and ED*” to systemic issues and “*real structures (government, capitalism, misogyny,* etc.*)*”, noting they felt that “*any approach that forgets this is doomed to fail. Part of the way to address food insecurity is to ensure it is eliminated*”. Involvement from clinicians in policy work was also suggested: “*I think mental health staff need to be more involved in policy solutions*” (P52, 29, F, White, ND).

Participants also wanted FI to be normalised by raising general awareness. For example, P272 (19, F, White, PD) stated they wanted “*Online platforms and more about the topic on the news to spread awareness and make people feel more comfortable around the topic rather than it being so secretive*”. Other participants wanted to challenge stereotypes about FI, for instance, P368 (38, F, White, PF) responded, “*Food insecurity also needs to be seen as a problem faced by everyone, I have BED therefore my body shape is not ‘typical’ of someone with an eating disorder or someone facing food insecurity. I find both topics difficult to approach because of my size and the fear of being judged, this is also why I don’t use food banks* etc.” Table 6 summarises all participant suggestions.

## 4. Discussion

This study examined the perspectives of PwEDs on the role of FI in the development, maintenance, and relapse of their illness, their experiences seeking help in clinical contexts, and their suggestions for clinical guidance. Quantitative analyses revealed that recent FI experiences in the current sample were related to self-reports of more frequent excessive exercise and fasting ED behaviours, less comfort discussing FI with a clinician, and being more adversely affected by multiple cost-of-living challenges, relative to participants without FI. Qualitative analysis illustrated that FI and the cost-of-living crisis were consistently reported to exacerbate restrictive and binge eating symptoms, and worsen quality of life. Those who experienced childhood FI believed it was linked to the onset of their ED. Recovery for participants with recent FI was also reportedly more difficult, with FI leading to a relapse of the ED in others. Help-seeking for FI was low in this sample, with shame, guilt, and embarrassment emerging as major barriers. Participants reported seeking help most frequently from family members; however, feelings of guilt and shame persisted in these cases. Participants also provided suggestions for clinical guidance which focused on community- and systemic-level changes.

In the current sample, 41.8% of participants reported experiencing at least one form of FI in the past month. This figure is elevated when compared to the national average (which ranges from 10 to 20% depending on the source [12,58,59]), as well as estimates from ED clinicians in the UK [47]. While this figure may be inflated by self-selection bias due to the wording of the study advertisement [60], previous research demonstrates that FI is two or even three times more prevalent in people with at least one mental health diagnosis or disability than those without [10,61]. Additionally, the specificity of the study advertisement has enabled the collection of a rich qualitative dataset in the current survey. Although untested in this sample, experiences of FI are likely to be acute in PwEDs from other vulnerable populations too (e.g., people from minoritized ethnic groups). These communities are known to also experience FI at higher rates [10], and are typically underserved in ED research and practice [62]. However, little is known about how co-occurring minority status may impact the relationship between FI and EDs. Future epidemiological studies (e.g., [63]) are necessary to characterize the extent of FI in ED populations and to understand how troubling health inequalities may be worse in certain groups. Moreover, indicators of FI in the current study came from a single-item question based on the US Department of Agriculture Food Security Survey [53]. FI experiences in people with EDs may be better characterized in future by using multiple items from psychometrically tested surveys. Inclusion of some measure of past FI experiences may also provide a better understanding of the chronology of FI indicators and ED course.

Cost-of-living concerns were rated as having a moderate to high impact on participants’ lives. This was especially true for participants with recent FI experiences. Nascent reports on this crisis suggest that, for instance, the inability to heat homes negatively impacts respiratory health in young children and the elderly [64], and financial uncertainty and stress related to increasing prices compromises mental well-being [16]. These outcomes are projected to worsen unless policy approaches are taken to mitigate the harm of the rising cost of living [65]. For PwEDs, the inability to afford heating their home is likely to be most detrimental to those with a low weight (e.g., those with AN or ARFID) who are particularly sensitive to feeling the cold [66]. Similar to what participants described in the sample, a ‘heat or eat’ dilemma—wherein individuals must choose between heating their homes or purchasing a sufficient amount of food—has been observed more widely as a consequence of the rising cost of living [67]. Vulnerable groups, such as single parents, also seem to bear the brunt of this, additionally feeling that they need to sacrifice their own food intake to ensure their children have nutritious diets [68]. Based on the descriptions from participants in the current sample, the added stress from the cost-of-living crisis is likely to worsen the symptoms of EDs.

Consistent with a social determinants framework [69], FI was described by participants as contributing to the development and maintenance of their ED. Participants who felt that FI contributed to their ED routinely reported that childhood experiences of FI created stressful scenarios around food which, in line with a life-course approach [70], later added to a poorer relationship with food. Participants also described a vicious cycle between FI, ED symptoms, and work and finances: (1) initial FI exacerbated ED symptoms, (2) the increased frequency and severity of ED symptoms hindered ones’ ability to work, and (3) being out of work decreased personal finances which led to more FI, wherein the cycle started again. In this sample, FI impacted the frequency of restrictive behaviours (such as excessive exercise or fasting due to weight/shape concerns), and qualitative descriptions from participants also suggested that FI worsened binge behaviours. These findings are partially inconsistent with previous research which found that FI was most often related to reports of binge episodes, and a greater likelihood that participants would meet the diagnostic criteria for EDs like BN and BED [34,35,36]. The relationship between FI and binging is largely predicated on the concept of the “feast/famine” cycle where intermittent access to food is expected to lead to binging during times of food abundance [37]. Differences in the current study findings may be related to the sample being mostly comprised of participants with experiences of AN. A floor effect may have been observed for binge/purge symptoms (i.e., objective binge episodes, self-induced vomiting, etc.) as the median scores for these variables were zero for both groups (Table 3). Interpretability of these findings is also limited as the current study did not include any control participants. Future research with more balanced diagnostic groups and controls might therefore find differences in these binging and purging symptoms. Additionally, the current results suggest, at the very least, that the link between restrictive symptoms and FI (which has thus far been underexplored in the research literature [37]) is also worth further investigation, and that a cogent mechanistic model is needed.

Help-seeking for FI was very low in the current sample, at only 7.7%. Accessible resources and familial support aided help-seeking; however, participants still routinely cited feelings of shame, guilt, and embarrassment when receiving help. Shame was the largest barrier for participants to seek help for FI. These findings are consistent with previous literature which shows that FI users of food banks in the UK report large amounts of social stigma related to this use [71]. Additionally, individuals experiencing FI are known to encounter feelings of shame related to their financial situation and difficulties accessing food [72]. The relationship between shame, stigma and lack of help-seeking are also well understood. Negative attitudes towards seeking professional help for mental health concerns are higher in those that feel ashamed about having a mental health condition [73]. Furthermore, experiences of shame and self-stigma have been consistently associated with reduced help-seeking for EDs, especially BED [74]. There is likely to be an interaction between shame and stigma derived from FI and poverty, and of that seen in EDs, which should be explored further.

Alongside low rates of help-seeking, participants reported rarely discussing FI with healthcare professionals (11%). This aligns with accounts from ED clinicians in the UK. Despite clinicians noticing a marked rise in FI among patients, they felt they lacked sufficient knowledge of how to intervene [47]. In the current study, suggestions for clinical guidelines included: (a) for FI to be asked about routinely at the assessment stage and for clinicians to initiate this (either via questionnaires or conversations); (b) for accommodations to treatment plans for patients experiencing FI; (c) for shame and embarrassment to be combatted by having healthcare professionals be empathetic and knowledgeable about FI and its interaction with ED symptoms; and d) to be provided with practical advice to alleviate their FI (i.e., local foodbank referrals, food voucher schemes, etc.). Our earlier clinician survey study also found that many ED healthcare practitioners were positive about many of these suggestions, including routine screening and psychoeducation about the interaction between FI and EDs. Additionally, previous attempts to alleviate FI by implementing money advice alongside psychological interventions in the NHS have been shown to be feasible and acceptable [75]. Plans to co-produce interventions for FI also exist in other populations, such as in pregnancy [76]. PwEDs may benefit from a similar attempt to co-produce an FI intervention that includes advice on managing finances, with other necessary resources. Ultimately, there is a dire need for directions for clinicians as participants reported that FI not only exacerbated symptoms, but that it also jeopardized their recovery. Specifically, FI impeded recovery efforts in some participants, while for others it precipitated a relapse of ED cognitions and behaviours. To raise awareness of the issues in clinicians, health care commissioners, policy makers, and other relevant stakeholders and to start addressing participants’ concerns, we have made a short animated film about FI and eating disorders (https://www.youtube.com/watch?v=lMhUHh4XpX8; accessed on 22 August 2025). We are also currently developing resources to aid ED clinicians in addressing FI in their practice, which we plan to disseminate nationally.

Participants also acknowledged that FI was a systemic problem that would require policy change to solve. A recent research briefing by the UK government acknowledged that the rise in the cost of living is affecting rates of FI and food bank use [58], while another report illustrates that household FI has increased since 2020, especially in low-income households and people with disabilities [77]. As discussed previously, current FI in the UK is likely related to austerity measures established and maintained by the UK government [15]. There have also been repeated calls for the government to implement critical policy changes to alleviate FI, with one article describing FI as a “public health issue left to fester” [14]. First steps towards policy solutions are being made. For example, the UK government is trialling the provision of free breakfasts before school in up to 750 schools, which may alleviate the burden of FI in some families. However, wider policy changes are still needed.

### Strengths and Limitations

The current survey has elucidated that FI plays a role in the development, maintenance, and relapse risk of EDs and is the first of its kind in the UK. The use of an online survey has enabled rich data to be collected across the UK and for a wide array of perspectives to be considered. However, this mode of data collection is not without its challenges. For example, the current study was reliant on participants’ self-report of previous and current ED diagnoses and symptoms, which could not be clinically validated due to the anonymous nature of the survey. Tools like the EDDS were implemented into the survey to provide information about probable diagnosis; however, as participants could end the survey early, this was not available for everyone. This limitation is likely to be present in future online survey studies of this nature, too. The cross-sectional design of the survey also limits the ability to draw any causal conclusions from the current research. Although the research literature suggests the relationship between FI and psychiatric conditions is likely bidirectional [27], longitudinal studies are necessary to properly parcellate these effects for PwEDs.

Additionally, participants in the current sample were largely white and female. As such, the generalisability of the current findings to minoritized ethnic and other PwEDs is questionable. As highlighted above, future research will need to delve into how minority status may impact the relationship between FI and EDs. Moreover, as efforts continue to make ED services more accessible to people from underserved communities, researchers will also need to consider the impact of this interaction on ED assessment and treatment. Lastly, as research develops in this field, it should consider weighting findings by the cost of living across UK regions. Inequities are usually concentrated in certain localities, so accounting for regional living costs may provide more nuanced information about which ED services are most likely to encounter referrals with co-occurring FI.

## 5. Conclusions

FI is a significant, yet understudied health issue for ED development and prognosis. Experiences of FI are quantitively and qualitatively associated with the maintenance of ED symptoms, and are antithetical to the stability of patients’ recovery. Stress induced by FI and the cost of living is likely to cause PwEDs to engage in ED behaviours, slow recovery efforts, and lead some people to relapse. Despite the magnitude of FI and its consequences, many PwEDs do not feel comfortable discussing struggles with accessing food with healthcare professionals, nor is screening of this issue routinely included in clinical practice. Further work is needed to train ED clinicians and other healthcare professionals on identifying FI, adapting protocols for treatment, and providing necessary resources to patients. Additionally, stigma at both the clinical and cultural levels needs to be addressed to allow PwEDs to more confidently discuss FI experiences and seek support when needed. However, the problem of FI will not be solved by clinicians alone, as it is related more widely to systemic issues like poverty, austerity, and the cost of living. As such, policy change is critical to effectively resolve the issue of FI.

## Figures and Tables

**Table 1:** Participant demographics and clinical characteristics.

Demographic Variable	Total Sample (*n* = 337)
Age	16–67 years (Mean = 27.78)
Gender	312 Female; 9 Male; 14 Non-binary/third gender; 2 self-described ^1^
Country of Residence	288 England; 37 Scotland; 11 Wales; 1 Northern Ireland
Employment Status ^2^	187 In Full-/Part-time Employment; 117 In Education; 29 Unemployed; 13 Temporarily Unemployed; 6 Prefer Not to Say; 6 Parent/Informal Carer; 1 Retired
Ethnicity ^3^	312 White; 12 Mixed or Multiple Ethnic Groups; 8 Asian; 3 Black; 2 Prefer Not to Say
Household Size ^4^	1–17 ^5^ people (Mean = 2.91)
Number of Dependents	0–4 (Mean = 0.44)
Parents/Carers	11.28%
Receive Social Assistance	28.19%
Co-occurring Mental Health Diagnoses	244 Yes; 91 No; 2 Prefer Not to Say
Currently Experiencing ED Symptoms	282 Yes; 48 No; 7 Prefer Not to Say
Currently or Previously in ED Treatment	224 Yes; 39 No
Frequency of ED Diagnoses ^6^	210 AN; 40 BN; 11 BED; 23 ARFID; 22 Other ^7^

^1^ Self-described as trans masc and neuroqueer; ^2^ Categories not discrete. “In Education” is inclusive of both school and university; ^3^ The full ethnic categories used in the survey are as follows: (1) Asian (e.g., Indian/Pakistani/Bangladeshi/Chinese/Asian British), (2) Black/African/Caribbean or Black British, (3) Mixed or multiple ethnic groups and (4) White (e.g., English/Welsh/Scottish/any other White background). Participants were given the option to specify their ethnicity if not included in this list; ^4^ Household size including participant; ^5^ Household size of 17 was reported by participant studying at university. This value may thus represent a university dorm; ^6^ Diagnoses are not discrete. 38 participants had multiple diagnoses. ARFID = Avoidant/Restrictive Food Intake Disorder; ^7^ Responses to “Other” include Eating Disorder Not Otherwise Specified (EDNOS), Other Specified Feeding or Eating Disorder (OSFED), and orthorexia nervosa.

**Table 2:** Descriptive statistics for dependent variables.

Dependent Variable	Mean (SD)
Food Insecurity Experience (*n* = 337) ^1^	
Worry about Affording/Accessing Food	118 (35.01%)
Eaten Less Nutritious/Balanced Meals	67 (19.88%)
Had Smaller Meals/Skipped Meals	65 (19.29%)
Been Hungry but Not Eaten	35 (10.39%)
Not Eaten for an Entire Day	12 (3.56%)
Eating Disorder Symptoms (EDDS) ^2^	
Fear of Weight Gain (*n* = 269)	5.14 (1.56)
Weight/Shape Concern (*n* = 269)	5.20 (1.38)
Frequency of Objective Binge Episodes (*n* = 269)	2.74 (4.22)
Frequency of Self-Induced Vomiting (*n* = 264)	2.02 (4.20)
Frequency of Laxative/Diuretic use (*n* = 265)	1.83 (4.15)
Frequency of Fasting (*n* = 265)	5.75 (5.76)
Frequency of Excessive Exercise (*n* = 264)	5.42 (5.66)
Global ED Symptoms (*n* = 205) ^3^	40.42 (19.40)
Cost-of-Living Experiences	
Rising Energy Costs (*n* = 280)	54.62 (30.26)
Affordability of Clothing (*n* = 286)	52.05 (32.52)
Rising Transport Costs (*n* = 284)	49.06 (32.08)
Reliance on Zero/Low Hour Contracts (*n* = 248)	20.14 (32.13)
Partake in Activities for Recovery (*n* = 289)	63.01 (29.85)
Time Off Work for Recovery (*n* = 277)	58.17 (38.21)

^1^ Participants were able to select all options that applied. Values outside of the parentheses for this variable are the frequency of each response; ^2^ The first 7 variables of the EDDS are from singular items on the scale (questions 2, 3, 6, 13, 14, 15, 16 respectively). The *n* for each item is variable because some participants chose to end the survey early; ^3^ There are two extra participants with Global ED symptom scores when compared to the Probable Diagnosis variable. This is because these participants did not provide information about their weight and/or their height, which is not used when computing the Global score.

**Table 3:** Inferential statistics for ED symptoms by recent FI experiences.

	Recent FI				No Recent FI				Inferential Statistics			
	*n*	Mean (SD)	Median	Mean Rank	*n*	Mean (SD)	Median	Mean Rank	*U*	*p*	*r*	Power (1 − β)
Eating Disorder Symptoms (EDDS)												
Fear of Weight Gain (*n* = 269)	125	5.11 (1.56)	6.00	131.87	144	5.16 (1.57)	6.00	137.72	8609	0.468	0.044	0.058
Weight/Shape Concern (*n* = 269)	125	5.30 (1.29)	6.00	139.00	144	5.10 (1.44)	6.00	131.52 ^1^	8499.5	0.359	0.056	0.236
Frequency of Objective Binge Episodes (*n* = 269)	126	2.51 (3.95)	0.00	133.58	143	2.94 (4.45)	0.00	136.26	8829.5	0.748	0.020	0.140
Frequency of SIV (*n* = 264)	123	1.87 (4.05)	0.00	131.40	141	2.16 (4.35)	0.00	133.46	8536.5	0.790	0.016	0.089
Frequency of L/D use (*n* = 265)	124	1.84 (4.10)	0.00	136.13	141	1.82 (4.20)	0.00	130.24	8353.5	0.413	0.050	0.050
Frequency of Fasting (*n* = 265) *	124	6.67 (5.77)	6.00	145.38	141	4.95 (5.65)	2.00	122.12 ^1^	7202.5	0.011	0.156	0.836
Frequency of ExE (*n* = 264) *	123	6.17 (5.78)	5.00	143.28	141	4.77 (5.48)	2.00	123.10 ^1^	7346	0.027	0.136	0.681
Global ED Symptoms (*n* = 205)	95	42.11 (18.71)	-	-	110	38.73 (19.95)	-	-	1.154 ^2^	0.250	0.162 ^3^	0.534

SIV = self-induced vomiting, L/D = laxative or diuretic, ExE = excessive exercise. Please see the Supplementary Materials for full details of scale ranges. * Significant at *p* = 0.05; ^1^ Variables did not have similarly shaped distributions between both groups, and so, the mean rank should be used to interpret findings; ^2^ Variable met assumptions for parametric tests. Value represents results from *t*-test with degrees of freedom = 203; ^3^ Value represents Cohen’s d for test.

**Table 4:** Inferential statistics for probable ED diagnosis by recent FI experiences.

	Recent FI		No Recent FI		Inferential Statistics	
Probable ED Diagnosis (EDDS)	*n*	%	*n*	%		
Dichotomous (*n* = 205)					χ^2^ (1) = 1.584, *p* = 0.208, OR = 0.620 (0.293, 1.311)	
Any ED Diagnosis	82	86.32	86	79.63		
No Current Diagnosis	13	13.68	22	20.37		
Within ED Diagnosis (*n* = 168)					Fisher’s exact = 4.548, *p* = 0.211, Cramer’s V = 0.170	
Anorexia Nervosa	59	71.95	58	67.44		
Bulimia Nervosa	19	23.17	17	19.77		
Binge Eating Disorder	0	0	4	4.65		
OSFED	4	4.88	7	8.14		

**Table 5:** Inferential statistics for comfort discussing FI and cost-of-living experiences by recent FI experiences.

		Recent FI				No Recent FI			Inferential Statistics			
	*n*	Mean (SD)	Median	Mean Rank	*n*	Mean (SD)	Median	Mean Rank	*U*	*p*	*r*	Power (1 − β)
Comfort Discussing FI (*n* = 325) **	138	44.41 (31.61)	47.00	139.44	187	58.54 (31.69)	61.00	180.39 ^1^	9651.5	<0.001	0.216	0.997
Cost-of-Living Experiences												
(a) Rising Energy Costs (*n* = 280) **	133	66.00 (29.54)	71.00	172.55	147	44.33 (29.26)	50.00	111.50 ^1^	5512.5	<0.001	0.377	>0.999
(b) Affordability of Clothing (*n* = 286) **	130	59.89 (32.15)	66.50	163.83	156	45.51 (31.57)	46.00	126.56 ^1^	7497	<0.001	0.225	0.995
(c) Rising Transport Costs (*n* = 284) **	130	55.92 (31.63)	59.00	160.21	154	43.27 (31.51)	46.50	127.55 ^1^	7708	<0.001	0.198	0.981
(d) Reliance on Zero/Low Hour Contracts (*n* = 248)	117	24.88 (36.24)	4.00	133.04	131	15.90 (27.55)	2.00	116.87 ^1^	6664.5	0.068	0.116	0.631
(e) Partake in Activities for Recovery (*n* = 289) **	133	75.83 (25.31)	82.00	181.68	156	52.08 (29.22)	50.00	113.73 ^1^	5495.5	<0.001	0.406	>0.999
(f) Time Off Work for Recovery (*n* = 277)	126	62.37 (38.75)	79.00	148.51	151	54.66 (37.66)	56.00	131.06	8314.5	0.069	0.109	0.525

** Significant at *p* <.001; ^1^ variables did not have similarly shaped distributions between both groups, and so the mean rank should be used to interpret findings.

**Table 6:** Participants’ suggestions for community- and systemic-level guidance.

Level	Guidance Area	Explanation and Examples
Community		
	ED Assessment	FI should be screened for routinely at the ED assessment stage. Clinicians should initiate discussions about FI, rather than waiting for patients to elect to reveal this information, regardless of the patient’s ethnicity, gender, diagnosis, disability status, or body size. FI should be assessed via questionnaires and/or discussions with patients. Care should be taken when broaching this subject, however, as many patients are likely to experience embarrassment about FI.
	ED Treatment	Once a clinician has identified that their patient experiences FI, adaptations to their treatment plans should be made. These may include providing budgeted meal plans, ideas for nutritious snacks, etc. Clinicians should be cognizant that many people experiencing FI are likely to be struggling financially in other areas, and that this may also impact their ability to implement treatment plans. Additionally, ED patients often experience difficulties with spending money and feelings of deservingness around food which are exacerbated by FI. Thus, these areas may be important targets for treatments.
	Practical Resources	Clinicians should not merely offer their commiserations; steps should be taken to provide practical resources to patients to help alleviate their FI. This may include information about how to access local foodbanks, voucher schemes, local charities, etc. Clinicians should avoid generic advice (i.e., batch cooking, buying in bulk, etc.) or just encouraging patients to look for this information themselves. This information can be made more widely available by placing it in common areas, like waiting rooms.
	Clinician Knowledge	Healthcare professionals (both primary and specialized) should receive training on FI. This should include, for example, how to identify FI, information on the ways that FI and different EDs may interact, and how FI may interfere with treatments, impede recovery, or facilitate relapse. Training should include perspectives of FI from those with lived experience of EDs.
	Reducing Stigma	FI can be more normalised in clinical settings by having posters around the hospital/practice which encourage patients to discuss their experiences of FI with their clinician. Placing psychoeducation materials about FI in common areas, such as waiting rooms, may also help. Empathy is necessary when discussing FI with patients as shame is a significant barrier to help-seeking.
Systemic		
	General Awareness	Public awareness of FI should be raised. Awareness campaigns should focus on helping people to identify indicators of FI, the impact of FI on physical and mental health, and it should encourage people to seek help if they are experiencing FI. They should also seek to tackle stereotypes about who can experience FI, such as those with higher body weight. It may be helpful to look to other successful awareness campaigns in the UK, such as those for safe driving or smoking, and to work with established charities and organisations (e.g., the Trussell Trust or Food Foundation) to effectively design campaigns.
	Policy	The systemic nature of FI should be at the forefront of public discussions. FI is unlikely to be solved just through clinical intervention efforts, and solutions to FI will require policy changes. Awareness campaigns should also focus on illustrating the harm of FI and the cost-of-living crisis to government bodies, and they should acknowledge the interaction between FI and other forms of marginalisation (e.g., ethnicity, gender, etc.). Advocacy from clinicians may be a necessary adjunct to lived experience perspectives.

## Data Availability

The data presented in this study are available on request from the corresponding author due to ethical restrictions.

## Supplementary Materials

The following supporting information can be downloaded at: https://www.mdpi.com/article/10.3390/nu18050852/s1, File S1: Survey of Food Insecurity and Eating Disorders.

## Abbreviations

The following abbreviations are used in this manuscript and are presented in order of use:

FI	Food insecurity
UK	United Kingdom (of Great Britain and Northern Ireland)
US(A)	United States (of America)
PwEDs	People with current or past eating disorders
COVID-19	Coronavirus Disease 2019
UN	United Nations
EDs	Eating disorders
BED	Binge eating disorder
BN	Bulimia nervosa
AN	Anorexia nervosa
NHS	National Health Service
*n*	Number of participants
ARFID	Avoidant/Restrictive Food Intake Disorder
EDNOS	Eating Disorder Not Otherwise Specified
OSFED	Other Specified Feeding and Eating Disorder
EDDS	Eating Disorder Diagnostic Scale
SPSS	Statistical Package for the Social Sciences
SD	Standard deviation
SIV	Self-induced vomiting
L/D	Laxatives/diuretics
ExE	Excessive exercise
PD	Previously diagnosed
ND	Not previously diagnosed
UD	Unknown diagnosis
F	Female
NB	Non-binary
M	Male
GP	General practitioner
CoL	Cost of living
N/A	Not applicable

## Appendix A

The number of responses to each question was variable. Table A1 shows the total number of useable responses to each question.

**Table A1:** Total number of responses to each qualitative question.

Question ^1^	Useable ^2^	No/Unsure ^3^	Unusable ^4^
Question 20 (*n* = 128)	128	-	-
Question 21 (*n* = 125)	90	33	2
Question 22 (*n* = 118)	102	-	16
Question 26 (*n* = 24)	11	-	13
Question 28 (*n* = 152)	137	9	6
Question 29 (*n* = 169)	145	20	4
Question 30 (*n* = 169)	139	26	4
Question 31 (*n* = 81)	55	14	12

^1^ Shortened versions of the questions can be found below. For full questions, see Supplementary Materials, File S1. Question 20: How has your experience of FI impacted your daily life (e.g., practically/emotionally)? Question 21: Are there any ways FI has contributed to the development of your ED symptoms? Question 22: How has FI impacted your ED and ED treatment (if applicable)? Question 26: Please describe your experience [of help-seeking for FI] (e.g., who and where, what help received). Question 28: Is there anything you would like to share about how the CoL crisis has impacted you (e.g., your daily life, mood, mental state, etc.)? Question 29: Do you have any thoughts on what such [clinical] guidance should include? Question 30: When thinking about discussing FI or the CoL with a healthcare professional, is the anything that would help you to do so? Question 31: Thinking about FI and your ED treatment, is there anything your therapist could do to help your recovery journey? ^2^ Number of responses that were useable in the analysis. ^3^ Number of responses, where appropriate to answering the question, where participants responded something to the effect of “No”, “Not sure”, “I don’t know” without elaborating. ^4^ Number of responses where it was either blank (e.g., responding “N/A” or “x”) or where they responded with unusable data.
