# Supplementary material for: Food Insecurity in Individuals with Eating Disorders: A UK-Wide Survey of Impact, Help-Seeking, and Suggestions for Guidance
Source: Nutrients. 2026 Mar 6;18(5):852. doi: 10.3390/nu18050852 (PMC12987311; doi:10.3390/nu18050852)
Supplement: Supplementary file 1 [file nutrients-18-00852-s001.zip › FI Paper Supplementary Materials_revised.pdf]

Supplementary Materials

File S1: Survey of Food Insecurity and Eating Disorders

The below contains the entire survey that was presented to participants via Qualtrics. The questions are presented as they were in the survey, including bold text for emphasis. An asterisk (*) next to a question denotes that the question was mandatory to proceed.

We would now like to ask a few questions about you.

Question 1*: Please enter your current age.

____

Question 2*: Which gender do you identify with?

- ○ Male
- ○ Female
- ○ Non-binary / third gender
- ○ Prefer not to say
- ○ Prefer to self describe: ______________________________

Question 3*: Where do you currently live?

- ○ England
- ○ Scotland
- ○ Wales
- ○ Norther Ireland
- ○ Other (please specify)*: ______________________________

Question 4*: Which of the following best describes your current education or employment status?

- ☐ At school or university
- ☐ Working full-time or part-time
- ☐ Parent or informal carer
- ☐ Not employed, short-term / looking for employment
- ☐ Not in education or employment
- ☐ Prefer not to say
- ☐ Other (please specify)*: ______________________________

Question 5*: Which ethnicity best describes you?

- ○ Asian (e.g. Indian / Pakistani / Bangladeshi / Chinese / Asian British)
- ○ Black / African / Caribbean or Black British
- ○ Mixed or multiple ethnic groups, or your specific ethnicity is not listed (Please specify)*:

  ____

- ○ White (e.g. English / Welsh / Scottish / any other White background)
- ○ Prefer not to say

Question 6*: How many people currently live in your household (including you)?

____

Question 7*: How many of those in your household are under the age of 18?

____

Question 8*: Are you a parent or informal carer (someone who provides unpaid help to a friend or family member needing support)?

- ○ Yes
- ○ No
- ○ Prefer not to answer

Question 9*: Are you currently in receipt of benefits or financial support from the UK government? (e.g., Universal credit)

- ○ Yes
- ○ No
- ○ Prefer not to answer

We will now ask you a few questions about your previous and/or current experiences with disordered eating or an eating disorder.

Question 10*: First we would like to know, have you ever been **diagnosed (by a professional) with an eating disorder**? For example, but not limited to, Anorexia Nervosa, Bulimia Nervosa, Binge Eating Disorder, Avoidant Restrictive Food Intake Disorder.

- ○ Yes
- ○ No
- ○ Prefer not to answer

Questions below only displayed if response to question 10 was "Yes".

Question 11: If you feel comfortable sharing, please let us know which diagnosis/es you received.

- ☐ Anorexia Nervosa (1)
- ☐ Bulimia Nervosa (2)
- ☐ Binge Eating Disorder (3)
- ☐ Avoidant Restrictive Food Intake Disorder (ARFID) (4)
- ☐ Other (please specify) (5) ____

Question 12*: Are you currently experiencing symptoms of your eating disorder?

- ○ Yes
- ○ No
- ○ Prefer not to answer

Question 13*: Are you currently in treatment, or have you ever received treatment for your eating disorder?

- ○ Yes (Please specify what kind of treatment you receive / have received)
- ○ No
- ○ Prefer not to answer

Question below only displayed if response to question 10 was "No" or "Prefer not to answer".

Question 14*: Have you ever struggled with symptoms or signs of an eating disorder, even though it was not clinically diagnosed?

- ○ Yes
- ○ No
- ○ Not Sure
- ○ Prefer not to answer

<u>Questions below only displayed if response to question 14 was "Yes" or "Not Sure".</u>

Question 15: If you feel comfortable sharing, please let us know which eating disorder symptoms you have experienced.

- ☐ Fasting / restricting as a means of controlling your weight or shape (1)
- ☐ Restricting or entirely avoiding certain foods or food groups for reasons **<u>other than</u>** controlling your weight or shape (2)
- ☐ Bingeing / overeating (feeling loss of control over eating, and eating far more than a person normally would at one go) (3)
- ☐ Purging, using laxatives, driven exercise or restriction as a means of controlling the effects of eating (4)
- ☐ Other eating disorder symptoms (please specify) (5)

____

Question 16: Are you currently experiencing these eating disorder symptoms?

- ○ Yes
- ○ No

Displayed for all:

Question 17*: Have you ever been diagnosed with a mental health disorder **<u>other than an eating disorder</u>**?

- ○ Yes
- ○ No
- ○ Prefer not to answer

<u>Question below only displayed if response to question 17 was "Yes".</u>

Question 18: If you feel comfortable sharing, please let us know which diagnosis/es you have received.

____

Your experiences with food insecurity

We will now ask you a few questions surrounding the topic of food insecurity and how it might relate to your eating disorder / eating disorder symptoms.

Food insecurity means the lack of regular access to enough safe and nutritious food for normal growth and development, and an active and healthy life. Food insecurity can arise from poverty (i.e., not being able to consistently afford food), or from not being able to access suitable foods (e.g., because of working or living conditions).

Food insecurity can be experienced at different levels of severity, ranging from uncertainty regarding the ability to obtain food to running out of food and going a day or more without eating:

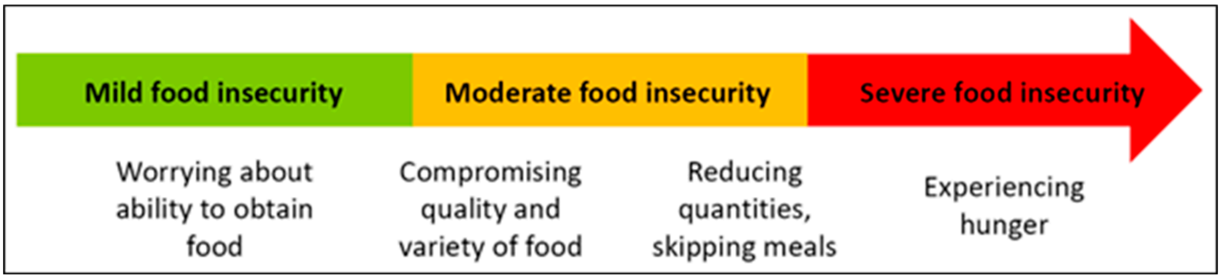

Source: Environmental Audit Committee, House of Commons, UK. Available at:https://tinyurl.com/2p9mpm93

Question 19*: **In the last month, have you or has anyone else in your household** *(please select all that apply)*

- ☐ Been **worried about accessing or affording** enough nutritious food?
- ☐ Had less nutritious or balanced meals **because you couldn't afford or get access to food?**
- ☐ Had smaller meals than usual or skipped meals **because you couldn't afford or get access to food?**
- ☐ Ever been hungry but not eaten **because you couldn't afford or get access to food?**
- ☐ Not eaten for a whole day **because you couldn't afford or get access to food?**
- ☐ None of the above

Questions 20-22 only displayed if participants selected any response to question 19 other than "None of the above".

Question 20*: Based on your responses, it seems that you have experienced food insecurity. How has your experience of food insecurity impacted your **day-to-day life?** We are interested

in both **practical** impacts (e.g., on how you choose to spend money) and **emotional** impacts (e.g., stress).

Question 21*: Are there any ways in which you feel food insecurity may have contributed to the **development** of your eating disorder symptoms, if at all?

Question 22*: How has your experience of food insecurity specifically impacted **your eating disorder and your eating disorder treatment** (if applicable)?

In the following questions, please keep in mind that food insecurity is a spectrum that ranges from worrying about being able to afford and access food, to experiencing hunger due to inability to afford or access food. All are significant, and we are interested in your experience wherever it lies on the spectrum.

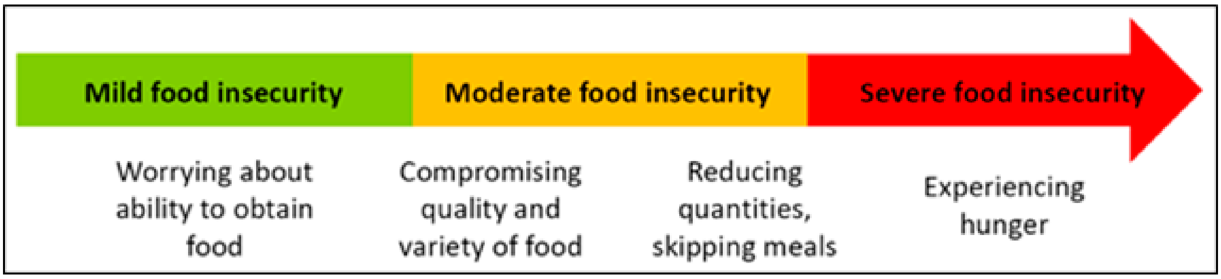

Question 23*: Has a healthcare professional (for example your GP or a therapist) ever asked you about food insecurity?

- ○ Yes
- ○ No
- ○ Prefer not to answer

Question 24*: How comfortable would you feel discussing the topic of food insecurity at an appointment with a healthcare provider?

Very Uncomfortable<br>0	Very Comfortable<br>100
---|---	

Question 25*: Have you ever sought help from anyone about food insecurity?

- ○ Yes
- ○ No
- ○ Prefer not to answer

Question below only displayed if response to question 25 was "Yes".

Question 26*: Please describe how that experience was for you. Where or who did you ask for help? How did it make you feel? What, if any, help did you receive?

____

Question 27*: In addition to food insecurity there are currently a number of **other cost-of-living issues** affecting many people in the UK. How much would you say do these following cost-of-living issues impact your day-to-day life in a practical sense?

	no impact 0 a great deal 100
a) Rising energy costs (e.g., affecting cooking and heating)	----------------------|----------------------
b) Affordability of clothes (e.g., when needing to change clothes size related to the eating disorder)	----------------------|----------------------
c) Rising transport costs (e.g., to attend in-person treatment sessions)	----------------------|----------------------
d) Reliance on zero hours or low-hours contracts	----------------------|----------------------
e) Ability to do other, non-food related things that are important for recovery / wellbeing (e.g., hobbies, outings, trying new things, socialising)	----------------------|----------------------
f) Taking time off work to focus on recovery	----------------------|----------------------

Question 28*: Is there anything you would like to share with us about how the **cost-of-living crisis more generally has impacted you personally**? This can be related to how you manage your day-to-day life, or your mood or your mental state (for example managing worries about health and the future, experiencing stigma or prejudice).

Your thoughts on resources and guidance on food insecurity and other cost-of-living issues for people with eating disorders and healthcare professionals

We are thinking of developing some resources for people with eating disorders and guidance for clinicians around food insecurity and cost-of-living issues.

Question 29*: Do you have any thoughts on what such guidance should include?

____

____

____

____

____

Question 30*: When you think about **discussing experiences of food insecurity** or **any cost-of-living issues** with a **healthcare professional** (for example your GP, your counsellor, your psychotherapist or psychiatrist), is there anything that would help you to do so?

____

____

____

____

____

Question 31 only displayed if participants selected any response to question 19 other than "None of the above".

Question 31*: Thinking about **food insecurity and your eating disorder treatment** in particular, is there something that your therapist could do to help you on your recovery journey?

____

____

____

Questionnaire about eating, exercising behaviour and perceived appearance

Thank you for completing the questionnaire.

Lastly, we will ask you a few questions about your thoughts, feelings and behaviours in relation to eating, exercising and body image. We are asking this to understand more about who has completed this survey and the types of eating-related difficulties they are facing at the moment.

* Please read each question carefully and answer as honestly as possible. There are no right or wrong answers.

Over the past 3 months

	0	1	2	3	4	5	6
1. Have you felt fat?	○	○	○	○	○	○	○
2. Have you had a definite fear that you might gain weight or become fat?	○	○	○	○	○	○	○
3. Has your **weight** or **shape** influenced how you think about (judge) yourself as a person?	○	○	○	○	○	○	○

4.* During the past 3 months, have there been times when you felt you have eaten what other people would regard as an unusually large amount of food (e.g., a pint of ice cream) given the circumstances?

- ○ Yes
- ○ No

5.* During the times when you ate an unusually large amount of food, did you experience a loss of control (feel you couldn't stop eating or control what or how much you were eating)?

- Yes
- No

6.* How many **times per month** on average over the **past 3 MONTHS** have you eaten an unusually large amount of food and experienced a loss of control?

- 0
- 1
- 2
- 3
- 4
- 5
- 6
- 7
- 8
- 9
- 10
- 11
- 12+

*** During these episodes of overeating and loss of control, did you**

	Yes	No
7. Eat much more rapidly than normal?	○	○
8. Eat until you felt uncomfortably full?	○	○
9. Eat large amounts of food when you didn't feel physically hungry?	○	○
10. Eat alone because you were embarrassed by how much you were eating?	○	○
11. Feel disgusted with yourself, depressed, or very guilty after overeating?	○	○
12. Feel very upset about your uncontrollable overeating or resulting weight gain?	○	○

* In order to prevent weight gain or counteract the effects of eating, how many **times per month** on average over the past **3 months**

	0	1	2	3	4	5	6	7	8	9	10	11	12	13	14+
13. ... have you made yourself vomit?	○	○	○	○	○	○	○	○	○	○	○	○	○	○	○
14. ... have you used laxatives or diuretics?	○	○	○	○	○	○	○	○	○	○	○	○	○	○	○
15. ... have you fasted (skipped at least 2 meals in a row)?	○	○	○	○	○	○	○	○	○	○	○	○	○	○	○
16. ... have you engaged in excessive exercise specifically to counteract the effects of overeating?	○	○	○	○	○	○	○	○	○	○	○	○	○	○	○

17.* How many **times per month** on average over the past **3 months** have you eaten after awakening from sleep or eaten an unusually large amount of food after your evening meal and felt distressed by the night eating?

- 0
- 1
- 2
- 3
- 4
- 5
- 6
- 7
- 8
- 9
- 10
- 11
- 12+

18.* How much does any eating and body image problem impact your relationships with friends and family, work performance, and school performance?

	0	1	2	3	4	5	6
	○	○	○	○	○	○	○

19.* How much do you weigh? Please specify **in kg**. If you are uncertain, please give your best estimate.

____

20.* How tall are you? Please specify **in cm**.

____

21.* What is your highest weight at your current height? Please specify in **kg**

____

Source Stice, E., Telch, C. F., & Rizvi, S. L. (2000). Development and validation of the Eating Disorder Diagnostic Scale: a brief self-report measure of anorexia, bulimia, and binge-eating disorder. Psychological assessment, 12(2), 123.

Question 32: Are there any other final thoughts you have about the topic of food insecurity and the cost-of-living crisis that you would like to share with us?

____

____

____

____

____
